# Assessment of Medial Gastrocnemius Thickness and Stiffness in Children with Obesity Using Shear Wave Elastography

**DOI:** 10.3390/children13101320

**Published:** 2026-09-28

**Authors:** Nurullah Dağ, İsmail Dündar, Şükrü Güngör, Sevgi Tasolar, Hilal Er Ulubaba, Rümeysa Akyol, Elif Damar Güngör

**Affiliations:** 1 Department of Radiology, Faculty of Medicine, Inonu University, 44280 Malatya, Türkiye; 2 Department of Pediatric Endocrinology, Faculty of Medicine, Inonu University, 44280 Malatya, Türkiye; 3 Department of Pediatric Gastroenterology, Faculty of Medicine, Inonu University, 44280 Malatya, Türkiye; 4 Department of Ophthalmology Clinic, Faculty of Medicine, Inonu University, 44280 Malatya, Türkiye

**Keywords:** childhood obesity, shear wave elastography, muscle mechanical properties, medial gastrocnemius muscle, metabolic risk

## Abstract

**Highlights:**

**What are the main findings?**

- Children with obesity had greater medial gastrocnemius thickness but lower shear wave elastography-derived stiffness than healthy-weight controls, indicating a dissociation between muscle size and mechanical properties.
- Lower muscle stiffness was associated with greater adiposity, insulin resistance, and adverse metabolic parameters.

**What are the implications of the main findings?**

- Shear wave elastography may provide information on skeletal muscle mechanical properties beyond conventional muscle thickness measurements in children with obesity.
- Assessment of muscle mechanical properties may help characterize obesity-related muscle impairment and its metabolic burden, although prospective studies with body-composition and functional reference measures are needed for validation.

**Abstract:**

Objectives: This study aimed to compare medial gastrocnemius (MG) muscle thickness and shear wave elastography-derived stiffness between children with obesity and healthy-weight controls and to examine associations with metabolic risk parameters. Methods: This cross-sectional study with prospective data collection included 92 children and adolescents, comprising 56 participants with obesity and 36 healthy-weight controls. Anthropometric, laboratory, and metabolic parameters were recorded. MG thickness and stiffness were assessed using standardized ultrasonography and shear wave elastography (SWE) protocols. Between-group comparisons were performed using the independent-samples *t* test or Mann–Whitney U test, as appropriate. Associations with metabolic parameters were evaluated using Pearson or Spearman correlation analysis, followed by partial correlation analysis adjusted for age, sex, and pubertal stage. Results: Children with obesity had lower MG stiffness than healthy-weight controls (16.80 ± 3.31 vs. 20.26 ± 3.02 kPa; *p* < 0.001) and greater MG thickness (2.37 ± 0.40 vs. 1.84 ± 0.38 cm; *p* = 0.023). After adjustment for age, sex, and pubertal stage, lower MG stiffness remained associated with higher BMI z-score (rp = −0.345, *p* = 0.001), waist circumference z-score (rp = −0.400, *p* < 0.001), WHtR z-score (rp = −0.359, *p* = 0.001), fasting insulin (rp = −0.296, *p* = 0.005), and HOMA-IR (rp = −0.315, *p* = 0.003). Conclusions: These findings indicate that SWE may add information to conventional ultrasonography when evaluating obesity-related alterations in skeletal muscle mechanical properties.

## 1. Introduction

Childhood obesity is often associated with a paradoxical increase in body weight but a relative reduction in functional lean mass, including skeletal muscle [1]. This may not be apparent from conventional anthropometric measurements alone. Identifying obesity-related changes in muscle properties at an early stage is important, as such changes may have long-term implications for metabolic health, physical performance, and quality of life [2,3].

Ultrasonography provides a practical approach for evaluating skeletal muscle morphology in children. In addition to conventional morphologic measurements, shear wave elastography (SWE) enables quantitative assessment of tissue mechanical properties by estimating muscle stiffness [4,5]. The medial gastrocnemius (MG) is particularly suitable for pediatric assessment because of its superficial location and functional relevance [6].

Skeletal muscle is the principal site of insulin-stimulated glucose disposal and has an important role in metabolic homeostasis [7,8]. In pediatric obesity, increased adiposity may be accompanied by qualitative skeletal muscle alterations, including intramyocellular or extramyocellular lipid accumulation, impaired insulin sensitivity, and reduced metabolic flexibility [9,10]. Conventional anthropometric measurements may not fully reflect these tissue-level changes. Therefore, SWE-derived MG stiffness may offer additional information on obesity-related muscle involvement and its relationship with metabolic risk [11,12].

The aim of this study was to compare MG muscle thickness and SWE-derived stiffness between children with obesity and healthy-weight controls, and to examine the associations of these measurements with anthropometric and metabolic risk parameters.

## 2. Materials and Methods

### 2.1. Ethical Approval and Study Population

This cross-sectional comparative study with prospective data collection was approved by the Institutional Ethics Committee (2025/7983), and written informed consent was obtained from the parents or legal guardians of all participants. The study was conducted in accordance with the principles of the Declaration of Helsinki. Eligibility was assessed among patients younger than 18 years who were evaluated at the Pediatric Endocrinology Outpatient Clinic between July and December 2025. Participants with a body mass index (BMI) above the 95th percentile for age and sex according to World Health Organization reference data were included in the obesity group. Age- and sex-matched healthy-weight children and adolescents with a BMI between the 5th and 85th percentiles were included as controls.

Exclusion criteria for both groups were syndromic or monogenic obesity; endocrine causes of obesity such as hypothyroidism, Cushing syndrome, or hypothalamic obesity; chronic systemic disease; neuromuscular disorders; previous major surgery affecting mobility; lower extremity muscle injury or surgery within the previous 6 months; and use of medications known to affect body composition, including systemic glucocorticoids or psychotropic agents.

### 2.2. Clinical, Anthropometric, and Laboratory Assessment

Age, sex, medical history, and pubertal status were recorded for all participants. Pubertal status was determined according to Tanner staging. Anthropometric measurements were obtained in the morning after an overnight fast, with participants wearing light clothing and no shoes. Height was measured to the nearest 0.1 cm using a Harpenden stadiometer (Holtain Ltd., Crymych, UK), and body weight was measured to the nearest 0.1 kg using a SECA digital scale (seca GmbH & Co. KG, Hamburg, Germany). BMI was calculated as weight in kilograms divided by height in meters squared and converted to age- and sex-specific z-scores using World Health Organization reference data [13]. Waist circumference was measured at the umbilical level during normal expiration. Waist circumference z-scores and waist-to-height ratio (WHtR) z-scores were then calculated. Blood pressure was measured by the auscultatory method using an appropriately sized cuff after at least 20 min of rest. Values above the 90th percentile for age, sex, and height were considered elevated.

Laboratory assessments were performed on venous blood samples obtained after an overnight fast. Fasting glucose, insulin, lipid profile, liver function tests, and thyroid function tests were analysed. Hyperinsulinaemia was defined according to pubertal stage: fasting insulin levels > 15 µIU/mL in prepubertal children, >30 µIU/mL in Tanner stages 2–4, and >20 µIU/mL in Tanner stage 5. Insulin resistance was calculated using the HOMA-IR formula, with values >2.5 in prepubertal children and >3.16 in pubertal children indicating insulin resistance [14]. Dyslipidaemia was defined as the presence of at least one of the following criteria: total cholesterol > 200 mg/dL, triglycerides > 150 mg/dL, LDL cholesterol > 130 mg/dL, or HDL cholesterol < 40 mg/dL [15].

Metabolic syndrome was assessed according to modified pediatric WHO-based criteria [16]. Participants in the obesity group were classified as having metabolic syndrome if, in addition to obesity, they had at least three of the following abnormalities: impaired glucose regulation; systolic blood pressure above the 95th percentile for age, sex, and height; hypertriglyceridaemia; and low HDL cholesterol according to age-specific reference values. Control participants were required to have laboratory parameters within the normal reference range and no clinical or biochemical findings suggestive of metabolic or endocrine disease.

Hepatic steatosis was assessed by ultrasonography and graded on a four-point scale (grade 0: absent, grade 1: mild, grade 2: moderate, and grade 3: severe) based on hepatic echogenicity, attenuation of the ultrasound beam, and visualization of intrahepatic vessels and the diaphragm [17].

### 2.3. Ultrasonography and Shear Wave Elastography Protocol

All ultrasonography and SWE examinations were performed by a third-year radiology resident under the direct supervision of a board-certified radiologist with 10 years of experience. Both were blinded to the participants’ clinical data. Interobserver and intraobserver agreement analyses were not performed. Examinations were performed using a single ultrasound system (Resona I9, Mindray, Shenzhen, China) equipped with a linear transducer (SC2-9 MHz). Example of greyscale US and SWE measurements are shown in Figure 1 and Figure 2. All examinations were performed with participants in the prone position, with the ankles in a neutral position and hanging freely over the edge of the examination table to maintain a passive muscle state. The MG muscle was evaluated at the proximal one-third of the line between the medial femoral condyle and the medial malleolus, corresponding to the region of the maximal muscle belly. Transverse B-mode scanning was first performed to identify the area of maximal MG thickness. The transducer was then rotated 90° to obtain longitudinal images for SWE acquisition. Muscle thickness was measured on grayscale US at the same level and recorded in centimetres (Figure 1). During SWE acquisition, the system’s built-in quality control indicators were used. Measurements were accepted only when the motion stability indicator (M-STB) showed 4–5 stars and a green signal level. On the reliability map (RLB-MAP), areas with a signal quality greater than 90% were selected whenever possible. For each measurement, a circular region of interest approximately 3–4 mm in diameter was placed in the mid-depth portion of the muscle. Care was taken to avoid excessive transducer pressure and visible muscle contraction. Measurements with poor signal quality were discarded and repeated. Five valid SWE measurements were obtained for each participant. Measurements were considered reliable when the interquartile range-to-median ratio was <0.30, based on the reliability parameters automatically calculated by the system. The median of the five measurements was recorded as the MG stiffness value in kilopascals (kPa) for each participant [18].

### 2.4. Statistical Analysis

Statistical analyses were performed using IBM SPSS Statistics for Windows, version 22.0 (IBM Corp., Armonk, NY, USA). Distributional assumptions were evaluated using the Shapiro–Wilk test. Continuous data are presented as mean ± standard deviation or median with interquartile range, according to distribution, whereas categorical data are summarized as frequencies and percentages. Between-group comparisons were performed using the independent-samples *t* test or Mann–Whitney U test for continuous variables and the chi-square or Fisher’s exact test for categorical variables. Bivariate associations between muscle ultrasound parameters and anthropometric or metabolic variables were evaluated using Pearson correlation for normally distributed variables and Spearman rank correlation for non-normally distributed variables. Pearson correlation coefficients were reported as *r*, whereas Spearman rank correlation coefficients were reported as *r*s. Partial correlation analyses were subsequently performed after adjustment for age, sex, and pubertal stage (Tanner stage), and the corresponding partial correlation coefficients were reported as *r*p. To avoid multicollinearity and model overfitting due to high inter-correlation among anthropometric markers (BMI z-score, waist circumference z-score, and waist-to-height ratio z-score), these covariates were analyzed in separate analytical frameworks. Multiple linear regression analyses were performed with muscle stiffness (kPa) as the dependent variable to evaluate its associations with anthropometric and metabolic parameters after adjustment for age, sex, and pubertal stage. Because BMI z-score, waist circumference z-score, and WHtR z-score were highly correlated, these variables were evaluated in separate models to minimize multicollinearity. Regression results are presented as unstandardized coefficients (B), standard errors (SE), standardized coefficients (β), and R^2^ values. A two-sided *p* value < 0.05 was considered statistically significant.

## 3. Results

### 3.1. Demographic and Clinical Characteristics

The study included 92 participants: 56 children and adolescents with obesity and 36 healthy-weight controls. The groups did not differ significantly in age, sex distribution, or pubertal status (all *p* > 0.05). The prevalence of hyperinsulinaemia, metabolic syndrome, dyslipidaemia, hepatic steatosis, impaired glucose tolerance or diabetes mellitus, acanthosis nigricans, striae, and subclinical hypothyroidism in the obesity group is shown in Table 1.

### 3.2. Anthropometric and Laboratory Parameters

Children with obesity had significantly higher waist circumference, waist circumference z-score, BMI z-score, and WHtR z-score than healthy-weight controls (all *p* < 0.001). Triglycerides, total cholesterol, uric acid, fasting insulin, HOMA-IR, HbA1c, liver size, and ALT were also significantly higher in the obesity group, whereas HDL cholesterol was significantly lower. No significant between-group differences were observed in LDL cholesterol, fasting glucose, AST, TSH, or vitamin D levels (all *p* > 0.05).

MG stiffness was significantly lower in children with obesity than in healthy-weight controls (16.80 ± 3.31 kPa vs. 20.26 ± 3.02 kPa; *p* < 0.001), whereas MG thickness was significantly higher in the obesity group (2.37 ± 0.40 cm vs. 1.84 ± 0.38 cm; *p* = 0.023) (Table 2).

### 3.3. Multivariable Analysis of Factors Associated with Muscle Stiffness

To evaluate factors associated with skeletal muscle mechanical properties, multiple linear regression analyses were performed with muscle stiffness (kPa) as the dependent variable. In separate models adjusted for age, sex, and pubertal stage, higher BMI z-score (β = −0.354, *p* = 0.001), HOMA-IR (β = −0.315, *p* = 0.002), waist circumference z-score (β = −0.410, *p* < 0.001), and WHtR z-score (β = −0.368, *p* < 0.001) were significantly associated with lower muscle stiffness (Table 3).

### 3.4. Correlation Between Elastography and Metabolic Syndrome Components

Partial correlation analyses were performed after adjustment for age, sex, and pubertal stage. Following covariate adjustment, muscle stiffness (kPa) maintained statistically significant inverse correlations with BMI z-score (r_p_ = −0.345, *p* = 0.001), fasting insulin (r_p_ = −0.296, *p* = 0.005), HOMA-IR (r_p_ = −0.315, *p* = 0.003), waist circumference z-score (r_p_ = −0.400, *p* < 0.001), and WHtR z-score (r_p_ = −0.359, *p* = 0.001). Additionally, muscle thickness preserved its significant positive associations with fasting insulin, HOMA-IR, hepatic steatosis grade, waist circumference z-score, and HbA1c. No significant adjusted correlations were observed between muscle stiffness (kPa) and triglyceride or HDL cholesterol levels (all *p* > 0.05) (Table 4).

Pearson correlation coefficients are denoted by r and Spearman rank correlation coefficients by rs. Adjusted coefficients are reported as rp. The correlation method used for each unadjusted analysis was selected according to the distribution of the variables.

## 4. Discussion

In this study, children with obesity had lower SWE-derived MG stiffness despite having greater MG thickness than healthy-weight controls. Lower MG stiffness was also associated with several adverse metabolic parameters, including higher adiposity indices, fasting insulin, HOMA-IR, and HbA1c. These findings suggest that muscle thickness and muscle stiffness may reflect different aspects of skeletal muscle involvement in pediatric obesity.

The emerging concept of obesity-related impairment in muscle health in children suggests that excess adiposity may coexist with adverse changes in muscle composition and function, potentially contributing to metabolic dysfunction [19,20]. Most previous pediatric studies have focused on anthropometric or body composition measures, whereas our findings suggest that mechanical muscle properties assessed by elastography may also be altered in children with obesity and may be associated with metabolic burden [21,22,23].

The combination of greater muscle thickness and lower stiffness suggests that increased muscle size should not necessarily be interpreted as preserved muscle quality. Enlargement of the muscle compartment in obesity may partly reflect non-contractile components, including intramuscular fat and extracellular tissue, whereas SWE provides information on the mechanical characteristics of the tissue itself [5,24]. Previous imaging studies have also demonstrated increased intramuscular fat and reduced muscle density in pediatric obesity [24,25]. Prior work similarly reports disproportionate muscle size gains without corresponding strength improvements in obese youth [26,27,28]. Thus, greater muscle size in obesity may coexist with altered mechanical properties and does not necessarily indicate preserved muscle function or composition.

Lower muscle stiffness was associated with several adverse metabolic features, including higher adiposity indices, fasting insulin, HOMA-IR, and HbA1c, whereas muscle thickness showed a less consistent pattern of associations. These findings are consistent with previous reports suggesting that skeletal muscle characteristics beyond muscle size alone may be relevant to cardiometabolic health [29,30]. Hepatic steatosis represents another relevant manifestation of metabolic dysfunction in pediatric obesity. According to the updated nomenclature, hepatic steatosis accompanied by cardiometabolic risk factors is classified within metabolic dysfunction-associated steatotic liver disease (MASLD), replacing the previous NAFLD terminology [31]. From a pathophysiological perspective, increased adiposity may impose greater metabolic demand, while altered muscle mechanical properties may reflect diminished metabolic reserve [32,33]. The regression analyses further supported these associations, showing that greater adiposity and insulin resistance were associated with lower MG stiffness after adjustment for age, sex, and pubertal stage. These findings support interpreting altered muscle stiffness as a tissue-level correlate of obesity-related metabolic alterations rather than as a diagnostic marker for obesity or metabolic syndrome.

The observed associations between lower MG stiffness and adverse metabolic parameters may reflect the broader interaction between skeletal muscle and systemic metabolic health in pediatric obesity. Skeletal muscle is a major site of insulin-mediated glucose disposal, and alterations in muscle composition and mechanical properties may accompany insulin resistance and increased adiposity. However, because muscle composition and functional performance were not directly assessed in the present study, these findings should be interpreted as associative rather than mechanistic. Further studies combining SWE with quantitative body-composition imaging and functional muscle assessment may help clarify the biological basis of these relationships [8,9,10].

SWE is noninvasive, radiation-free, relatively rapid, and feasible in pediatric practice. Unlike modalities that primarily estimate muscle quantity, SWE provides quantitative information on tissue mechanical properties and may offer additional insight into obesity-related muscle alterations [17,34]. The medial gastrocnemius is particularly suitable for pediatric assessment because of its superficial location and functional relevance in weight-bearing activity [6,35]. From a pediatric imaging perspective, SWE may serve as a practical adjunct to conventional ultrasonography when evaluating obesity-related muscle changes in children.

This study has several limitations. Its cross-sectional design precludes causal inference, and the assessment was limited to a single muscle group. Reference-standard measures of muscle composition and muscle function were not available, limiting mechanistic interpretation. SWE is device- and operator-dependent, and pediatric reference values remain limited. Subcutaneous adipose tissue thickness at the measurement site was not recorded, and interobserver or intraobserver reproducibility was not assessed. In addition, physical activity and participation in structured sports were not systematically assessed across all participants and therefore could not be included as covariates in the multivariable analyses. Information on dietary interventions and obesity-related treatments was also not systematically available. Finally, the relatively small sample size may have limited subgroup analyses and the detection of smaller effects.

## 5. Conclusions

Children with obesity demonstrated increased MG thickness accompanied by lower SWE-derived stiffness. The association between reduced stiffness and adverse anthropometric and metabolic parameters suggests that SWE may complement conventional ultrasonography by providing additional information on obesity-related changes in skeletal muscle mechanical properties.

## Figures and Tables

**Figure 1:**
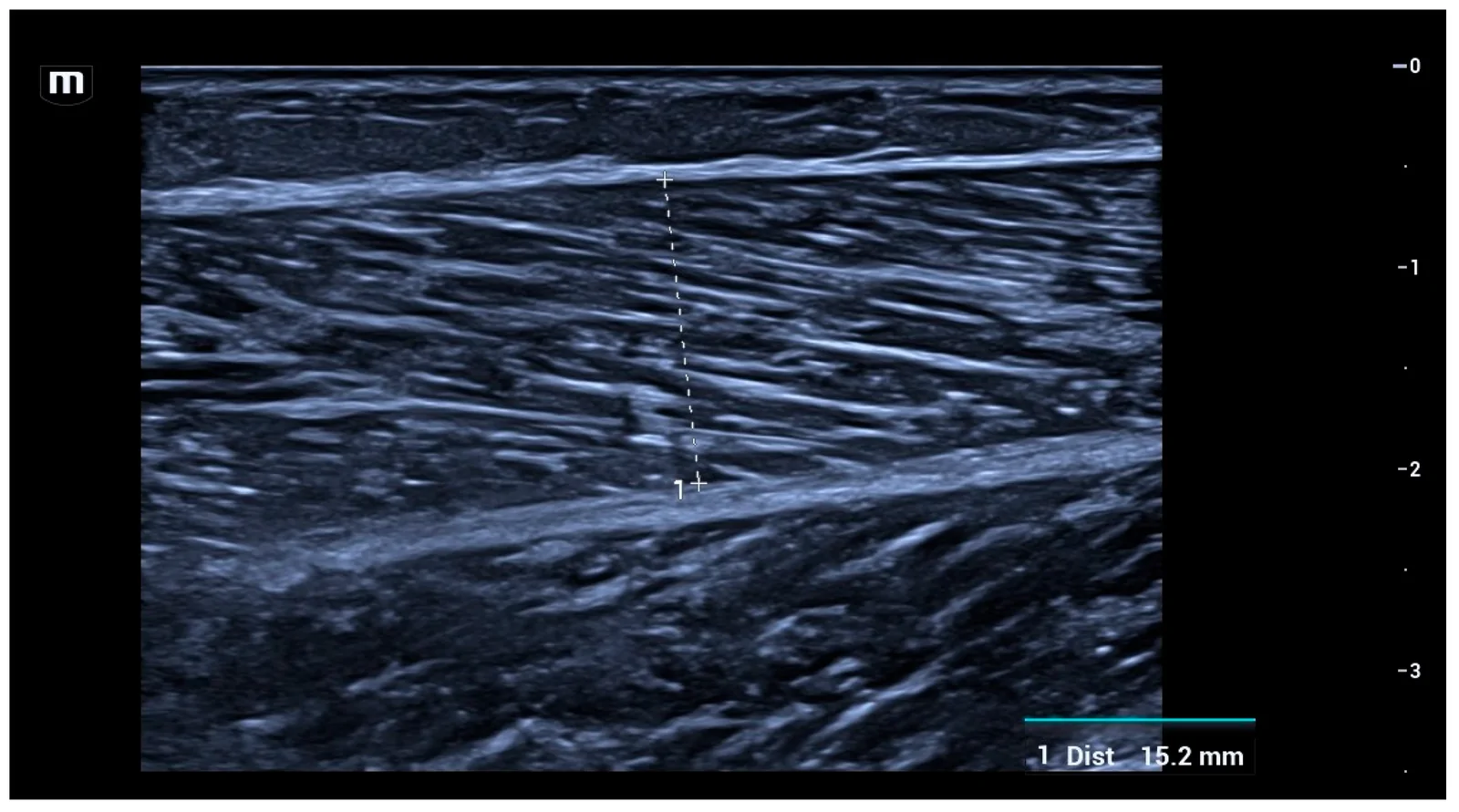
Grayscale ultrasonography of the medial gastrocnemius muscle demonstrating thickness measurement.

**Figure 2:**
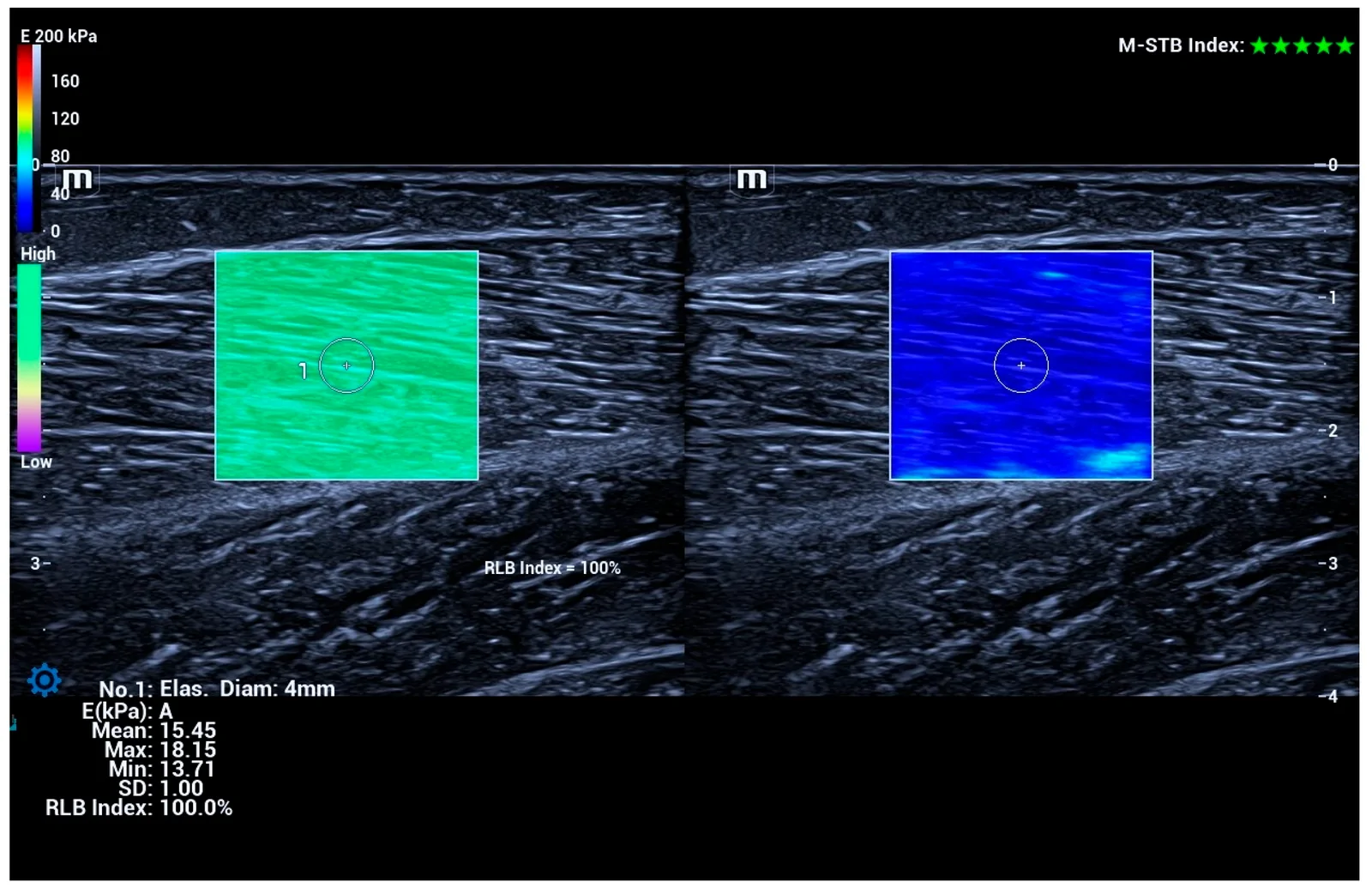
Shear wave elastography (SWE) evaluation of the medial gastrocnemius muscle with reliability map (RLB) and elastography stiffness map. The left panel shows the reliability map (RLB; green area) indicating optimal signal quality (RLB Index: 100%). The right panel displays the corresponding SWE stiffness map with a circular ROI placed in the mid-muscle region. Quantitative stiffness parameters (mean, min–max, SD) are automatically generated using a standardized 4-mm ROI under high motion stability (M-STB: 5 stars).

**Table 1:** Demographic and clinical findings of participants.

Variable	Obese (N = 56), *n* (%)	Healthy (N = 36), *n* (%)	*p*-Value
**Sex**			0.689
Female	35 (62.5%)	21 (58.3%)	
Male	21 (37.5%)	15 (41.7%)	
**Pubertal Status**			0.180
Prepubertal	13 (23.2%)	13 (36.1%)	
Pubertal	43 (76.8%)	23 (63.9%)	
**Hyperinsulinemia**	23 (41.1%)		
**Metabolic Syndrome**	23 (41.1%)		
**Dyslipidemia**	23 (41.1%)		
**Hepatic Steatosis**	30 (53.6%)		
**Prediabetes/Diabetes Mellitus (IGT/DM)**	19 (33.9%)		
**Acanthosis Nigricans**	22 (39.3%)		
**Striae**	25 (44.6%)		
**Subclinical Hypothyroidism**	2 (3.6%)		

**Table 2:** Clinical and laboratory findings of participants.

Variable	Obese (N = 56), Mean ± SD	Healthy (N = 36), Mean ± SD	*p*-Value
**Age (years)**	12.77 ± 3.43	12.34 ± 3.37	0.562
**Waist circumference (cm)**	96.28 ± 20.03	64.00 ± 7.30	<0.001
**Waist circumference Z-score**	1.74 ± 0.53	−0.43 ± 0.40	<0.001
**BMI Z-score**	2.65 ± 0.76	0.18 ± 0.70	<0.001
**Triglycerides (mg/dL)**	117.00 ± 48.20	88.23 ± 18.26	<0.001
**Total cholesterol (mg/dL)**	158.00 ± 30.84	143.00 ± 21.94	0.006
**LDL cholesterol (mg/dL)**	94.65 ± 28.72	91.70 ± 31.16	0.828
**HDL cholesterol (mg/dL)**	46.94 ± 10.85	51.00 ± 7.08	0.035
**Fasting glucose (mg/dL)**	84.43 ± 7.80	83.89 ± 7.24	0.740
**Fasting insulin (µIU/mL)**	21.52 ± 9.74	6.12 ± 2.85	<0.001
**HOMA-IR**	4.45 ± 2.16	1.28 ± 0.64	<0.001
**WHtR Z-score**	1.69 ± 0.58	−0.58 ± 0.67	<0.001
**HbA1c (%)**	5.36 ± 0.57	5.07 ± 0.29	0.006
**Liver size (cm)**	14.77 ± 1.58	11.65 ± 0.97	<0.001
**Uric acid (mg/dL)**	5.20 ± 1.11	3.75 ± 1.09	<0.001
**AST (U/L)**	27.62 ± 17.05	24.55 ± 7.52	0.312
**ALT (U/L)**	28.85 ± 21.33	16.72 ± 7.36	<0.001
**Free T3 (pg/mL)**	3.98 ± 0.55	4.27 ± 0.88	0.050
**Free T4 (ng/dL)**	0.85 ± 0.14	0.94 ± 0.16	0.007
**TSH (µIU/mL)**	2.76 ± 1.33	2.31 ± 1.56	0.137
**Vitamin D (ng/mL)**	17.23 ± 13.19	17.19 ± 10.31	0.991
**Muscle stiffness (kPa)**	16.80 ± 3.31	20.26 ± 3.02	<0.001
**Muscle thickness (cm)**	2.37 ± 0.40	1.84 ± 0.38	0.023

**Table 3:** Multivariable Linear Regression Models for Muscle Stiffness (kPa).

Models	Independent Predictors	Unstandardized B (SE)	Standardized β	t-Value	*p*-Value	R^2^
Model 1	BMI Z-score	−0.901 (0.261)	−0.354	−3.447	0.001	0.132
Model 2	HOMA-IR	−0.521 (0.160)	−0.315	−3.261	0.002	0.120
Model 3	Waist Circ. Z-score	−1.270 (0.306)	−0.410	−4.145	<0.001	0.176
Model 4	WHtR Z-score	−1.044 (0.286)	−0.368	−3.643	<0.001	0.144

Muscle stiffness (kPa) served as the dependent variable (outcome) across all models. All models were adjusted for age, sex, and pubertal stage. Abbreviations: B: unstandardized regression coefficient; SE: standard error; β: standardized regression coefficient; BMI: body mass index; HOMA-IR: homeostatic model assessment of insulin resistance; WHtR: waist-to-height ratio.

**Table 4:** Unadjusted and Adjusted Correlations Between Muscle Ultrasound Parameters and Metabolic Variables.

Variables	Muscle Stiffness (kPa), Unadjusted Correlation	Muscle Stiffness (kPa), Adjusted *r*p	Muscle Thickness, Unadjusted Correlation	Muscle Thickness, Adjusted *r*p
BMI Z-score	−0.348 (*p* = 0.001)	−0.345 (*p* = 0.001)	0.186 (*p* = 0.075)	0.197 (*p* = 0.066)
Fasting Insulin	−0.313 (*p* = 0.002)	−0.296 (*p* = 0.005)	0.267 (*p* = 0.010)	0.233 (*p* = 0.029)
HOMA-IR	−0.331 (*p* = 0.001)	−0.315 (*p* = 0.003)	0.291 (*p* = 0.005)	0.265 (*p* = 0.013)
Hepatic Steatosis	−0.089 (*p* = 0.400)	−0.074 (*p* = 0.495)	0.310 (*p* = 0.003)	0.278 (*p* = 0.009)
Waist Circ. Z-score	−0.417 (*p* = 0.001)	−0.400 (*p* < 0.001)	0.247 (*p* = 0.018)	0.242 (*p* = 0.023)
WHtR Z-score	−0.374 (*p* = 0.001)	−0.359 (*p* = 0.001)	0.222 (*p* = 0.034)	0.196 (*p* = 0.067)
HbA1c	−0.212 (*p* = 0.042)	−0.203 (*p* = 0.058)	0.268 (*p* = 0.010)	0.231 (*p* = 0.030)
ALT	−0.124 (*p* = 0.240)	−0.090 (*p* = 0.405)	0.210 (*p* = 0.045)	0.179 (*p* = 0.094)
Triglycerides	0.201 (*p* = 0.059)	0.193 (*p* = 0.071)	−0.233 (*p* = 0.028)	−0.017 (*p* = 0.875)
HDL-Cholesterol	0.122 (*p* = 0.254)	0.116 (*p* = 0.280)	0.047 (*p* = 0.662)	−0.138 (*p* = 0.198)

## Data Availability

The datasets generated and/or analyzed during the study may be obtained from the corresponding author upon reasonable request. Public deposition was not undertaken because of participant privacy and ethical requirements.
